# An Inspectorate Perspective on Serious Youth Violence and Criminal Exploitation

**DOI:** 10.3390/bs16040478

**Published:** 2026-03-24

**Authors:** Oliver Kenton, Robin Moore, Andrea Brazier, Helen Mercer, Helen Davies

**Affiliations:** HM Inspectorate of Probation, Manchester Civil Justice Centre, Manchester M3 3FX, UK; andrea.brazier@hmiprobation.gov.uk (A.B.); helen.mercer@hmiprobation.gov.uk (H.M.); helen.davies@hmiprobation.gov.uk (H.D.)

**Keywords:** violence, exploitation, safeguarding, knife crime, safety, youth justice, inspection

## Abstract

HM Inspectorate of Probation is committed to building and utilising the evidence base for high-quality youth justice services, and to promoting excellence and having a positive impact upon those inspected and the wider sector. Research evidence and inspection findings are used to inform understanding of what helps and what hinders services and to consider system-wide change. In this article, the latest inspection and research findings in relation to the high-profile areas of serious youth violence and criminal exploitation are highlighted. The article encompasses insights from core and thematic inspections, including those from recent joint targeted area inspections (JTAIs) undertaken with other inspectorates. Alongside the JTAIs which examined multi-agency responses to serious youth violence, research was commissioned to hear directly from children and families about their experiences. Other research commissioned and published by the Inspectorate has emphasised the importance of implementing relational, child-centred and trauma-informed approaches and to optimising collaborative/partnership working across agencies and sectors. Reports have also drawn attention to the value of paying attention to the socio-ecological framework, systemic resilience, adultification biases, and both contextual and transitional safeguarding.

## 1. Introduction

His Majesty’s Inspectorate of Probation (HMI Probation) promotes excellence in probation and youth justice services across England and Wales. Through its statutory mandate, its independent status, and the expertise and experience of its inspectors, the Inspectorate provides a distinct and influential voice on the quality and effectiveness of services. To help drive high-quality delivery, the Inspectorate undertakes independent inspections, produces recommendations, commissions and undertakes research, and publishes effective practice guidance. As well as inspections of individual and regional services, regular thematic reports are published on key issues in the criminal justice system, which includes joint thematic inspections to maximise the knowledge and skills held across differing inspectorates, resulting in authoritative and evidence-informed judgements and advice. Two such joint thematic inspections have focused upon multi-agency responses to serious youth violence (2024) and criminal exploitation (2018).

The HMI Probation research and data analysis function provides further evidence to maximise learning, with leading academics commissioned to provide their views on a range of relevant topics, recognising the value to be gained from differing perspectives and sources of evidence. From 2022 to 2026, papers have been published on the topics of serious youth violence, masculinity, gangs, county lines, child criminal exploitation, collective safeguarding, systemic resilience, adultification biases, and professional curiosity. Over the same period, the Inspectorate has published findings from primary research projects on knife crime and both contextual and transitional safeguarding.

Key themes from across all these publications—inspections, research and insights—are the focus of this paper. Recent high-profile cases involving children and young people, including fatal incidents such as the murders of Ronan Kanda in 2022 and Elianne Andam in 2023 (Home Office, 2025), have underlined the importance of identifying and implementing evidence-informed approaches. It is vital that agencies optimise their service delivery and the support provided to children and young people, minimising the risk of future serious incidents and delivering positive outcomes for both children and wider society.

## 2. Materials and Methods

This article highlights the key findings from HMI Probation’s inspections and research on serious youth violence and related topics such as contextual safeguarding (which recognises the contextual dynamics of extra-familial harm), transitional safeguarding (an approach to safeguarding adolescents and young adults fluidly across developmental stages), child criminal exploitation (CCE), and knife crime. Joint targeted area inspections (JTAIs) undertaken with the Office for Standards in Education, Children’s Services and Skills (Ofsted), the Care Quality Commission (CQC), and His Majesty’s Inspectorate of Constabulary, Fire and Rescue Services (HMICFRS) are critical sources. A 2024 report examined multi-agency responses to serious youth violence (Ofsted et al., 2024), while a 2018 report examined the multi-agency response to older-child exploitation and children missing from home, care or education (Ofsted et al., 2018).

Alongside these inspections, the article integrates the evidence from relevant Academic Insights papers and Research and Analysis Bulletins published by HMI Probation from 2021 to 2026. The Inspectorate’s Research and Analysis Bulletins present findings in relation to individual research projects, while the Academic Insights papers present the views of leading academics on specific topics, assisting with informed debate and aiding understanding of what helps and what hinders the delivery of high-quality youth justice services.

## 3. Findings

Looking across the inspections and commissioned/published research and insights, a number of key themes emerge for the provision of high-quality services and delivery. This section highlights the importance of the following:

1. Understanding the scale of serious youth violence, knife crime, and CCE.
2. Understanding the local context.
3. Understanding the contexts and needs of individual children.
4. Recognising the full range of safeguarding issues.
5. Implementing relational, child-centred and trauma-informed approaches.
6. Optimising collaborative/partnership working.
7. Focusing upon young adults as well as children.
8. Using and developing the evidence base.

### 3.1. Understanding the Scale of Serious Youth Violence, Knife Crime, and CCE

HMI Probation’s youth justice annual report for 2021 reported that children involved with serious youth violence made up a larger and growing proportion of the statutory caseload of those services inspected (HM Inspectorate of Probation, 2022). Recent youth justice statistics also confirm an increase in the proportion of proven offences by children falling within the ‘violence against the person’ category, rising from 24% in 2015 to 34% in 2024 (Youth Justice Board, 2026). In the 2024 Joint Targeted Area Inspection (JTAI) on multi-agency responses to serious youth violence, inspectors found that the extent and impact of serious youth violence was more pervasive than is often recognised by local services. For some specific offences, such as child-to-parent domestic abuse, the stigma is such that under-reporting is likely, leading to unaddressed risks of violence to both parents and children (Holt, 2022).

In terms of knife crime, public and political concerns have grown over recent years, with the number of teenagers murdered with a knife having more than doubled since 2013/2014 (Youth Endowment Fund, 2025a). Many children, including some as young as 11, report carrying knives because they feel unsafe and see knife carrying as a way of protecting themselves, failing to recognise the associated risks (Gray et al., 2021). Worryingly, carrying a knife has become normal to many of these children (Phillips et al., 2022b; Ofsted et al., 2024), further evidenced within the 2025 *Trends in violence affecting children report* (Youth Endowment Fund, 2025a). The 2025 thematic inspection on diversion and out-of-court disposals (given to children to address offending behaviour without the involvement of the courts) found that knife crime was an issue in the child’s life in many of the cases inspected and that this was not always considered where the offence itself was not knife crime-related (HM Inspectorate of Probation and HM Inspectorate of Constabulary and Fire & Rescue Services, 2025).

In terms of CCE, the Jay Report indicates that the number of identified children has been increasing, up to 14,420 in the year ending March 2023, compared to 10,140 in the previous year. Similar increases were seen in referrals to the National Referral Mechanism—the national framework for identifying and referring victims of modern slavery, of which CCE is considered a subset. These increases were in part due to an increase in awareness and identification, but also due to a real increase in prevalence (Jay et al., 2024).

### 3.2. Understanding the Local Context

Place-based approaches, which draw from socio-ecological theory, recognise the vital importance of understanding local context, issues and needs for an effective approach to serious youth violence (Pitts, 2021). Within JTAIs, more effective multi-agency and partnership work has been found to take place when serious youth violence has become an established strategic priority, enabling a shared understanding of local need. Partners in these areas have used consultations with communities, families and children to inform their work, alongside analysis of relevant information (Ofsted et al., 2024).

There were similar findings in the 2018 JTAI, which focused on criminal exploitation, human trafficking and modern slavery. It was emphasised that everyone involved needed to understand local issues of exploitation and gangs so that responses could be carefully coordinated to meet local need. Partnerships needed to not only identify and respond to the risk of exploitation, but work with children, parents and local communities to prevent exploitation through awareness-raising (Ofsted et al., 2018). The report states that “local partnerships need to be aware of the risks of exploitation in their local area. They must be curious at a strategic and operational level about what is happening in their locality” (Ofsted et al., 2018, p. 19). More recently, it has been acknowledged that the lack of national data has made it difficult to identify areas with the most significant concerns about CCE, while the lack of integration between local systems has made it difficult to share data and keep multiple partners up to date (Jay et al., 2024).

### 3.3. Understanding the Contexts and Needs of Individual Children

Understanding the full context of a child’s life is vital for a personalised approach, recognising that each child’s strengths and challenges will be different, shaped by their own characteristics, experiences, and circumstances. A recent systematic review of UK qualitative research with children involved in serious youth violence has highlighted the roles of trauma, poverty, school exclusion, and hypermasculinity (Barker et al., 2025). Needs related to neurodiversity and special educational needs and/or disabilities (SEND) are common. As identified within HMI Probation’s research on identifying safety concerns relating to children (K. Ball et al., 2022), the concerns relating to the children themselves and to other people are often overlapping and intertwined, with links to carrying knives or other weapons; illegal drug possession; drug and alcohol misuse; adversity and trauma; care experience; criminal exploitation, including county lines (Pitts, 2021); mental health issues; domestic abuse; family issues; and negative peer influences (K. Ball et al., 2022).

Similarly, the 2025 diversion and out-of-court disposals thematic inspection found that many of the children whose cases were examined had experienced adverse childhood experiences (ACEs), which encompassed witnessing domestic violence and experiencing neglect, abuse, discrimination, and bullying (HM Inspectorate of Probation and HM Inspectorate of Constabulary and Fire & Rescue Services, 2025). These experiences and their impact on children’s development had not always received adequate attention. There were concerns around exploitation in 42 of the 96 cases examined, but these were acknowledged and addressed appropriately in fewer than half, leaving some children at continued risk (HM Inspectorate of Probation and HM Inspectorate of Constabulary and Fire & Rescue Services, 2025).

An important component of a personalised and contextual approach is to apply a gendered lens, recognising that girls involved in the youth justice system can face additional vulnerabilities. In the thematic inspection on diversion and out-of-court disposals (HM Inspectorate of Probation and HM Inspectorate of Constabulary and Fire & Rescue Services, 2025), the girls in the inspection sample were much more likely to have committed a violent offence (73% of girls in the sample compared to 39% of boys; similar differences can be seen in the 2024 to 2025 youth justice statistics (Youth Justice Board, 2026)). They were twice as likely to have care experience compared to boys and were more likely to have unmet gender-related needs, including how their gender influenced their experience of trauma, exploitation, and coercion. They were less likely to be in mainstream school and more likely to have an education, health and care plan (EHCP; targeted at children who need more support than can be provided by special educational needs support). Inspectors emphasised the importance of a gender-responsive approach rather than relying upon universal services (HM Inspectorate of Probation and HM Inspectorate of Constabulary and Fire & Rescue Services, 2025), recognising that due to the disproportionate number of boys in the youth justice system, universal services tend to be tailored towards boys.

At the same time, a gender-responsive approach is required to account for the specific circumstances and needs of boys. It has been highlighted that many boys within the youth justice system have internalised damaging ideals that equate masculinity with control, aggression and emotional suppression, with these constructions underpinning behaviours linked to violence, domestic abuse and sexual harm (King-Hill, 2026). Through understanding these linkages and applying a gendered lens, practitioners can interpret behaviours accurately and address root causes rather than symptoms, challenging harmful narratives and reframing masculinity to reject violence and promote empathy, emotional literacy and healthy relationships.

Other protected characteristics require attention. There is an ethnic/race dimension, with serious youth violence disproportionately experienced by those from specific ethnic backgrounds (Youth Endowment Fund, 2025a). Agencies also need to be mindful of the age of the child. For some groups of children, such as those from a Black ethnic background, those involved with the care system and those who are reluctant to engage with services, notions of innocence and vulnerability are more likely to be displaced by notions of responsibility and culpability for their actions—a process called adultification—which can leave them more vulnerable (Maxwell, 2024; Davies, 2022).

### 3.4. Recognising the Full Range of Safeguarding Issues

While serious youth violence poses obvious risks from perpetrators to others (the latter often being other children), there are also significant risks to the children who display violent behaviour. The 2024 JTAI (Ofsted et al., 2024) found that too often, the risk of violence was not identified as a safeguarding issue, leaving the child in question at risk. Furthermore, because there is no comprehensive government guidance on how extra-familial harm should be managed, Local Safeguarding Partnerships and many agencies were not focusing sufficiently on safeguarding outside the family.

Research conducted for HMI Probation on the extent to which contextual safeguarding has been adopted by youth justice agencies supports the position that services are not yet fully responding to the contexts around extra-familial harm for children (Firmin et al., 2023). This is echoed in research with children and their parents undertaken for the 2024 JTAI, where parents reported that children’s social care services struggled to assess risk outside the home, such as at school or in the neighbourhood, or even recognise that it could be a problem (Safer London, 2024). Crucially, these omissions were hindering access to appropriate support for criminally exploited children (Jay et al., 2024).

The 2018 JTAI on protecting children from criminal exploitation, human trafficking and modern slavery (Ofsted et al., 2018, p. 19) highlighted the importance of focusing upon initial identification of those at risk of exploitation; “when a child presents with offending, or other concerning behaviour, professionals need to be curious and compassionate and ask: what is happening in this child’s life that is causing them to behave this way?”. This kind of enquiry should be prioritised by all involved agencies, with practitioners aware that even children not known to services or not stereotypically at risk might be vulnerable to exploitation (Jay et al., 2024; Maxwell, 2024).

### 3.5. Implementing Relational, Child-Centred and Trauma-Informed Approaches

The research literature has increasingly stressed the importance of holistic and collaborative child-centred and trauma-informed approaches within youth justice (see, for example, Day & Malvaso, 2025). In research commissioned by the Inspectorate which focused upon knife crime and promising approaches to mitigating this problem (Phillips et al., 2022b), knife crime programmes were perceived as useful, but they were not seen as sufficient on their own—they needed to be considered as part of a broader package of individualised and trauma-informed interventions. Effective partnership working has been identified across the JTAIs, addressing the wider needs of children affected by serious youth violence. The best-performing services understood the individual needs and histories of the children they worked with, including their experience of trauma and abuse. Effective initiatives focused on addressing the impact of abuse, helping children to access education, giving children opportunities to develop interests and skills, and helping them to stay safe (Ofsted et al., 2024).

The need to be trauma-informed is further highlighted by the research findings in relation to ACEs. For those within the youth justice system, early ACEs are common and lead to children being neurologically locked into a flight, fight or freeze response, often defaulting to “fight” as a way of protecting themselves, even when there is no threat present (Gray et al., 2021). Trauma-informed ways of working have been described as changing the perspective from “‘What’s wrong with you?’ to ‘What happened to you?’” (Gray et al., 2021, p. 4). The concept of Systemic Resilience is also relevant; bringing together systemic thinking and resilience theory, it locates the onus for developing resiliency within the web of relationships around children (Chard, 2022). A focus is required upon strengthening protective factors including within their families and their communities, and in the services that are available.

The 2018 JTAI on protecting children from criminal exploitation, human trafficking and modern slavery found that children were often groomed or threatened and not always able to see or accept that they had been exploited. The report stresses that professionals should not give up on such children, or their families, but should focus on communication and building relationships, recognising the risks of further violence and the need for help, support and protection (Ofsted et al., 2018). The JTAI-accompanied research highlights how failures to establish relationships can lead to persisting inaccurate perceptions, resulting in parents and their children feeling misrepresented and blamed, and available services not being accessed (Safer London, 2024).

To personalise delivery and ensure that children receive the appropriate holistic support at the right time, careful consideration also needs to be given to the youth justice disposals used. Out-of-court disposals—which are typically used to intervene early in a child’s offending behaviour—are seen as an appropriate way to avoid costly court appearances and the potential stigmatising effects of entry into the full youth justice system. However, the 2025 thematic inspection on diversion and out-of-court disposals found that the non-statutory, discretionary, police-led Outcomes 20 and 21 were not always used appropriately. Outcome 20 is a code used to close a case because action is being undertaken by another agency, typically the child’s school, while Outcome 21 is a code used to close a case because prosecution is not in the public interest. In the thematic inspection, it was found that these approaches were being used for some serious offences, including serious cases involving rape and offences involving weapons (HM Inspectorate of Probation and HM Inspectorate of Constabulary and Fire & Rescue Services, 2025). This was linked to inconsistent use of the Child Gravity Matrix (a triage tool developed by the National Police Chiefs’ Council which produces a score to indicate a suitable disposal) by police officers in multi-agency safeguarding hubs or when working as school officers.

The generally unmonitored use of Outcome 20 cases and the lack of involvement of the local youth justice service meant that concerning patterns of behaviour were not being identified and addressed through tailored holistic support. It is notable that since the thematic inspection, government guidance for child knife possession offences has been published, stating that police officers should alert the youth justice service of knife possession offences within one working day or as soon as practicable, and that these offences should only be met with a charge, a Youth Conditional Caution or a Deferred Prosecution (HM Government, 2026).

### 3.6. Optimising Collaborative/Partnership Working

Inspectors have emphasised how collaborative and partnership working between statutory agencies, education and voluntary sectors can reduce the risk of serious youth violence, building trust with local communities to identity and support those with whom they work (Ofsted et al., 2024). Similarly, the 2018 JTAI on protecting children from criminal exploitation, human trafficking and modern slavery was clear that “the only way of responding to and preventing highly organised criminal operations that exploit children is to have a highly coordinated multi-agency and whole-council approach” (Ofsted et al., 2018).

The wider research literature has also stressed that effective adolescent safeguarding systems require effective relationships and collaborations within and between multi-agency and multi-disciplinary partnerships (E. Ball & McManus, 2024). It has been argued that at its best, adolescent safeguarding involves the adoption of a relational approach at every level of the local system. There must be continual and active investment in these relationships, and it should not be assumed that it will be an “automatic by-product” from the legal requirement of safeguarding being “everyone’s responsibility” (E. Ball & McManus, 2024, p. 9).

While JTAIs have highlighted examples of local partnerships undertaking effective work to reduce harm to children from serious youth violence, inconsistencies in practice and the support available for children have also been identified. In 2022, the Home Office published the Serious Violence Duty, which obligates councils and local services to work together to share information and deliver interventions to prevent and reduce serious violence (Home Office, 2022). However, despite the expectations set out within the duty, not all areas have been found to have access to violence reduction units (VRUs—non-statutory, collaborative partnerships designed to build local capacity to reduce violence) and the additional resources they can provide, and not all of the VRUs were found to be making a positive difference to children (Ofsted et al., 2024).

**Figure 1:**
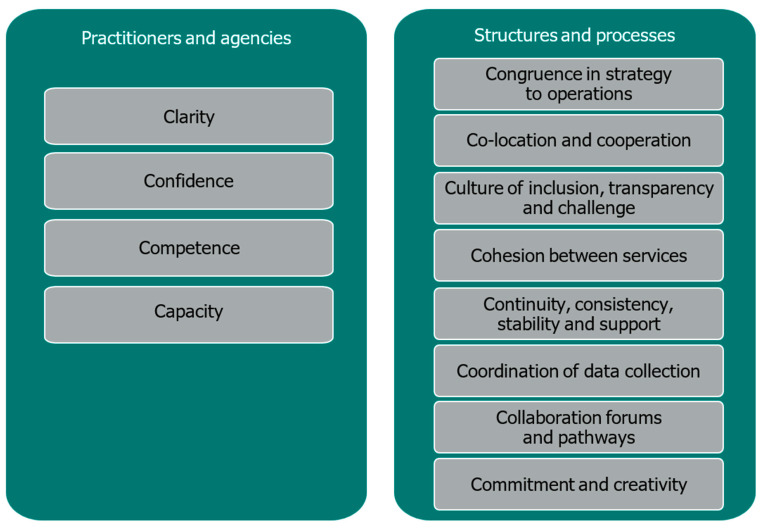
The 12Cs for collective safeguarding (reproduced from E. Ball & McManus, 2024).

In relation to safeguarding children, the ‘12Cs’ Collective Safeguarding Responsibility Model has been developed to encourage a more a consistent approach to measuring and evidencing multi-agency practices, encompassing good practices and challenges, with clear accountabilities and a shared commitment to future improvements (E. Ball & McManus, 2024). As set out in Figure 1, the model outlines 12 key components which facilitate effective multi-agency safeguarding practice, encompassing distinct yet interlinked issues relating to ‘practitioners and agencies’ and those relating to ‘structures and processes’. The key aim is to facilitate and operationalise the transition from safeguarding being ‘everyone’s responsibility’ to an accountable ‘collective responsibility’.

In 2022 the Inspectorate published research examining the approaches being adopted by youth justice services in response to knife crime (Phillips et al., 2022b). Across those services included in the research, it was found that they were keen to adopt a public health approach, taking the partnership approach to a more strategic level and facilitating agencies in being more proactive and preventative in tackling violence in the community (Local Government Association, 2018). Commissioning arrangements, funding, and strategic remits were seen as the main challenges, with two of the five areas having made more progress—these services were involved at a higher strategic level in developing preventative services in partnership with other agencies (Phillips et al., 2022b). The consequences of short-term funding, notably how it hinders the ability of agencies to make long-term plans, have also been highlighted through JTAIs (Ofsted et al., 2024).

### 3.7. Focusing upon Young Adults as Well as Children

The age of majority in the UK is 18. After an individual’s 18th birthday, they are treated legally as adults, and with the label of ‘adult’ comes the expectation to act in an adult manner. However, treating adolescence as covering the period from 10 to 24 years of age corresponds more closely to adolescent development. Brain development, particularly of the pre-frontal cortex (and its links with the limbic system, which manages, amongst other things, consequential decision making), can continue into the mid-twenties (House of Commons Justice Committee, 2016). The effects of incomplete development and maturity can be profound and can leave young adults more sensitive to emotional triggers and more likely to engage in risk-taking and aggressive behaviour. Notably, criminal and violent behaviours tend to peak in late adolescence and early adulthood and then decline steadily with age (Youth Endowment Fund, 2025b).

Children involved in offending behaviour are supervised by their local youth justice service, but supervision in the community in relation to offending once aged 18 is undertaken by the probation service (HM Inspectorate of Probation, 2024). This transition occurs at a period of peak offending for some individuals, and can break up supportive and trusting relationships with youth justice service practitioners which have taken time to establish. The transition between youth and adult probation services coincides with other transitions away from youth services that might also be supporting the young person, such as movement from children’s homes, children’s social care, and college education (Cocker et al., 2024). Various statutory services, such as mental health services, also have higher thresholds for involvement with adults than for children, and the young person thus faces losing wide-ranging potential and actual support, often at the time when they need it most.

The cliff edge of support at age 18 is well recognised within the sector and has implications for both supervision and safeguarding, particularly for those involved with serious youth violence. The Inspectorate’s thematic inspection examining the quality of services delivered to young adults in the Probation Service (HM Inspectorate of Probation, 2024) found that although this age group were particularly at risk of serious violence, there were shortfalls in delivery:

- There were challenges in accessing information from the police, making it more difficult to conduct comprehensive risk and safeguarding assessments and to plan to manage those risks.
- Checks with domestic abuse units focused on the young adult’s responsibility for domestic abuse or whether they had been a victim, but rarely considered whether there were wider domestic abuse concerns.
- Checks with social services were often not conducted or did not ask the right questions, despite nearly half the inspected cohort having prior involvement with social services in relation to abuse, neglect, criminal exploitation, or violent households.

**Figure 2:**
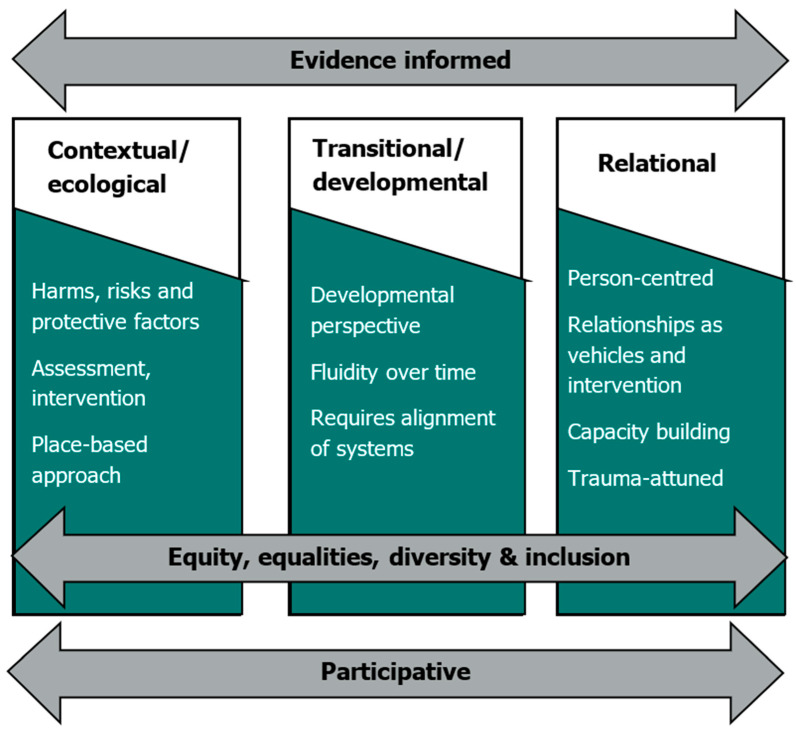
The six key transitional safeguarding principles (reproduced from Cocker et al., 2024, p. 55).

The research evidence highlights the potential value from introducing more fluid services across the transitional age range or, at the very least, closer collaboration between adult and youth services, with well-organised multi-agency arrangements and clear lines of responsibility (Cocker et al., 2024). Notably, the concept of transitional safeguarding promotes a more fluid non-binary approach to safeguarding as young people transition to adulthood. A holistic framework is applied, underpinned by six interconnected and interdependent principles (see Figure 2). These principles highlight how effective practice with both children and young adults is relational, participative and developmentally attuned, with consideration given to the individual, interpersonal, community, and societal levels within the social–ecological framework.

Research commissioned by HMI Probation found that transitional safeguarding is not yet fully embedded in probation or youth justice practice, despite the recognition by senior leadership of its value and benefits (Cocker et al., 2025). Probation was described as less flexible and having fewer resources than youth justice services, and consequently less able to work contextually. Young adults were also more likely to struggle with adult services when they were experienced as less supportive in relation to issues such as neurodiversity, transportation access, and housing.

### 3.8. Using and Developing the Evidence Base

Across the areas inspected as part of the 2024 JTAI, it was found that services were not sufficiently using or contributing to the evidence base around serious youth violence. There was insufficient ongoing evaluation of the effectiveness of work to address violence and insufficient sharing of effective practice across areas (Ofsted et al., 2024). At the wider policy level, it has been further highlighted that there needs to be a greater use of collated data and evaluations of new approaches. Various resources are available to assist with this work, such as the UK Government’s Test, Learn and Grow programme, and the Nesta test and learn playbook (Vojtkova et al., 2025).

The value of knowledge partnerships in youth justice has also been recognised, enabling the sharing of ideas, insights and experiences between members of the research/academic community and youth justice professionals (Creaney & Price, 2025). Through aligning the worlds of academia/research and practice, the partnerships can be mutually beneficial, bridging gaps between evidence and action while also facilitating the further development of the evidence base (Creaney & Price, 2025). More generally, it has been stressed that leaders need to focus upon building cultures of evaluation and continuous learning, with staff given sufficient time and space to continually reflect and learn, promoting an environment in which everybody feels responsible for evidence-informed and evidence-based practice (Moore et al., 2025).

## 4. Discussion

**Figure 3:**
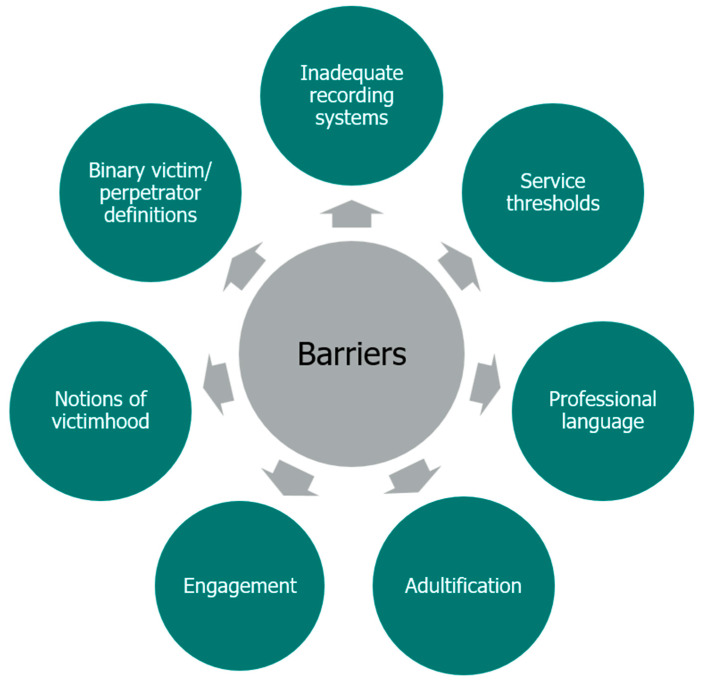
Barriers to protecting criminally exploited young people (reproduced from Maxwell, 2024).

The prevalence of serious youth violence, knife crime, and CCE remains highly concerning, with significant safety concerns and risks to the children involved and those around them. Within the Inspectorate’s research on identifying safety concerns, the following were all identified as issues requiring attention: inadequate assessments and recording, a lack of professional curiosity, insufficient focus on contextual safeguarding, a lack of planning for future risks and adverse outcomes, and a failure to spot changing risk when reviewing a case (K. Ball et al., 2022). At a broader level, service responses have been hindered by various structural and systemic factors; Maxwell (2024), for example, has highlighted the seven barriers to protecting criminally exploited young people, which are set out in Figure 3.

Adopting a child-centred, trauma-informed, and strengths-based approach can be seen as particularly important given that exploitation removes the young person’s control over their own life. The social–ecological framework is helpful, highlighting the need to understand the child in the context of their life and responding in a holistic and child-centred way, paying attention to the individual, interpersonal, community, and societal levels (Kemshall & McCartan, 2022). An intersectional lens can also be applied, exploring the potential impacts from race/ethnicity, sexuality, class, gender, (dis)abilities, and wider lived experiences (Davies, 2022). Understanding all relevant factors and how they interact can help to build a more holistic view of safety (encompassing physical, relational and psychological safety) and the full range of safeguarding issues, identifying which children are most likely to require assistance and improving the targeting of interventions. The higher the number of risks and the lower the number of strengths and protective factors, the more likely that a broad package of holistic and collaborative intervention will be required.

Critically, serious youth violence and CCE need to be understood as complex, cross-cutting issues, with the support required for the children involved typically too complex for any single agency, service, organisation, or professional sector to provide alone. It is essential to develop effective systems of cooperation between services to ensure that children can access the resources most needed through well-developed and integrated services and pathways (Viding et al., 2025). Agencies must work together through a shared language, an understanding of each other’s roles and responsibilities, and systems that enable collaborative working and the sharing of critical data. Practitioners need access to specialised multi-agency training and appropriate safeguarding tools (E. Ball & McManus, 2024), and they need to exercise professional curiosity, looking beyond the visible evidence and questioning why a young person is behaving in concerning ways. Exercising professional curiosity is not straightforward, and attention needs to be given to addressing any structural, relational and emotional barriers to its enactment. Notably practitioners need: (i) time and space to ask the right questions, analyse and act; (ii) time and space to develop relationships with children; and (iii) emotional support, recognising the emotional labour linked to its employment (Phillips et al., 2022a).

Engagement is one of the potential barriers identified in Figure 3. Criminally exploited young people may be reluctant to engage with professionals due to a culture of distrust of professionals, negative experiences with professionals, or fear that they will be taken into local authority care, be arrested, or be seriously harmed by the people exploiting them. This places the onus on professionals to work with young people to establish trust and to create safe spaces for them to share their experiences. The importance of trusting relationships cannot be understated; the children require adults who are persistent, reliable and consistent in their relationships. They need to know that the practitioner is not deterred by challenging behaviour, that the practitioner is on their side, and that the relationship requires no form of payback.

As also highlighted in Figure 3, there is a need to think carefully about language. Professional language accumulated over years has not always been receptive to considering children as victims of CCE, with language such as “making choices” and “putting themselves at risk” taking the focus away from their exploitation and towards a more responsibilising approach (Maxwell, 2024). It must be recognised that language is rarely neutral and it can be powerful, impacting upon thoughts and actions, and it is important to ensure that positive relationships are not hindered and opportunities for positive development are not lost.

Another barrier identified in Figure 3 is that of service thresholds. Viding et al. (2025) argue that attention should be given to breaking down barriers between local agencies and partners, and reducing high thresholds that might lead to young people being turned away from support. As has been set out in this article, there are particular issues when a young person turns 18. The transition from youth to adult probation services coincides with transitions away from other supportive youth services, with various statutory services having higher thresholds for involvement for adults than for children. Young people thus face losing wide-ranging potential and actual support, often at the time when they need it most.

## 5. Conclusions

Providing the necessary support to children and young people where there are concerns in relation to serious youth violence and/or CCE is far from straightforward. However, the work of HMI Probation and its companion inspectorates and partnered academics has shown that there are well-researched frameworks and approaches that can be adopted to help mitigate the risks and support positive outcomes. Understanding of the local context, issues and needs is essential, with a strong focus upon identification of the affected children, even when the offending behaviour is difficult to detect or the children themselves do not feel at risk of violence or exploitation. Once identified, understanding the full context of a child’s life is vital, recognising that each child’s strengths and challenges will be different, shaped by their own characteristics, experiences, and circumstances. Contextual safeguarding, which involves paying attention to sources of extra-familial harm and the contexts and people in their lives, must replace a perspective that views safeguarding as a predominantly intra-family issue. Personalised, relational and child-centred approaches—utilising a broad range of trauma-informed and evidence-informed interventions to address the specific needs of each child—must be used. Such practice necessitates the collaborative engagement of multiple agencies and partners from youth justice services to education, health and local community organisations, recognising that the holistic support required is typically too complex for any single agency, service, organisation, or professional sector to provide alone.

Finally, services and partnerships need to continuously research and evaluate their approaches and interventions, and share the results on a wide scale. Positive outcomes are more likely when practice is aligned to evidence, and the evidence base should continually evolve, enabling delivery to be improved over time and ultimately maximising positive outcomes for individual children and wider society.

## Data Availability

No new data were created or analysed in this study. Data sharing is not applicable to this article.
